# Diagnostic and Therapeutic Approach in ANCA-Associated Glomerulonephritis: A Review on Management Strategies

**DOI:** 10.3389/fmed.2022.884188

**Published:** 2022-06-03

**Authors:** Adél Molnár, Péter Studinger, Nóra Ledó

**Affiliations:** Department of Internal Medicine and Oncology, Semmelweis University, Budapest, Hungary

**Keywords:** ANCA-associated vasculitis, glomerulonephritis, microscopic polyangiitis (MPA), granulomatosis with polyangiitis (GPA), autoimmune kidney disease

## Abstract

Anti-neutrophil cytoplasmic autoantibody (ANCA)-associated vasculitis is a destructive small vessel vasculitis affecting multiple organs. Renal involvement often leads to end-stage renal disease and increases mortality. Prompt diagnosis and initiation of adequate immunosuppressive therapy are critical for the best patient and kidney outcomes. However, considerable heterogeneity in symptoms and severity across the patients frequently hinder the diagnosis and management. The objective of this review is to emphasize the heterogeneity of the ANCA-associated vasculitis, facilitate the recognition and give guidance to the therapeutical possibilities. We present epidemiologic and risk factors, pathogenesis, and provide comprehensive clinical features of the disease. This article also focuses on the currently available therapeutic options and emerging cellular and molecular targets for the management of systemic and especially renal disease. We conducted extensive literature research published on PubMed and Google Scholar. We systematically reviewed, analyzed, and assembled databases, covering a broad spectrum of aspects of the disease. We compared and summarized the recommendations of two recent guidelines on ANCA-associated vasculitis. The incidence of ANCA-associated vasculitis, hence glomerulonephritis shows a steady increase. Familiarity with the presenting symptoms and laboratory abnormalities are necessary for rapid diagnosis. Early initiation of treatment is the key aspect for favorable patient and renal outcomes. A better understanding of the pathogenesis constantly leads to more targeted and therefore more efficient and less toxic treatment.

## Introduction

Anti-neutrophil cytoplasmic antibody (ANCA)-associated vasculitis (AAV) is an inflammatory disorder of the small arteries, resulting in vascular destruction and tissue necrosis affecting various organs (1). The major clinicopathological categories of AAV are microscopic polyangiitis (MPA), granulomatosis with polyangiitis (GPA), and eosinophilic granulomatosis with polyangiitis (EGPA). According to serology, anti-myeloperoxidase (aMPO) and anti-proteinase 3 (aPR3) positive diseases are distinguished.

Glomerulonephritis is a frequent manifestation of AAV, presenting in almost all cases of MPA and frequently in GPA (2, 3). Renal involvement is of particular importance since it affects renal and overall patient morbidity and mortality (4). Despite advances in treatment, renal involvement in AAV still bears high mortality (3, 4). Therefore, early recognition and treatment can decrease the disease burden on patients and healthcare costs.

## Prevalence

The worldwide reported prevalence of AAV ranges from 4.6 to 21.8 cases per one million person-years (5–7). Most studies agree that its incidence increases over time (5, 8, 9). However, the rise might be explained by better recognition and more widespread availability of the ANCA test (10, 11).

There is an obvious geographical difference between the distributions of MPA and GPA; MPA is more frequent in Southern Europe and Asia, while GPA is more prevalent in Northern Europe and Australia (5, 9, 12). The geographical distribution of the serotypes is partially explained by the ambient ultraviolet radiation levels and latitude but also proposes a heterogeneity in the genetic background (13). The average onset of AAV is 65 years and prevalence peaks in the 70–75-year age group (7, 12, 14), but it is not limited to adults (15, 16). Patients with renal involvement are 10 years older on average compared to those without it (4). Though AAV displays a slight overall male predominance (8, 9, 17–19), renal involvement is more often present in females (4, 14, 20), which may be explained by the female predominance of MPO-AAV and its higher incidence with glomerulonephritis. PR3-AAV, on the other hand, is associated with male gender, a younger age, and a higher glomerular filtration rate at the diagnosis (13).

## Pathogenesis

Meta-analysis and genome-wide association studies (GWAS) confirm different genetic background for MPO- and PR3-AAV (21, 22).

According to the current hypothesis, AAV occurs in genetically predisposed patients who were exposed to certain environmental factors. GWAS identified several human leukocyte antigen (HLA) and non-HLA regions associated with AAV. GPA is linked to HLA-DP1, α1-antitrypsin (SERPINA) and proteinase 3, MPA to HLA-DQ, and EGPA to HLA-DRB4. Single-nucleotide polymorphism in protein tyrosine phosphatase (PTPN22), cytotoxic T lymphocyte-associated antigen-4 (CTLA4), Toll-like receptor 9, Fc gamma receptors, interleukin-10 and 2 were also associated with higher occurrence of AAV (23, 24). Environmental risk factors include certain medications, silica dust, organic solvents, and most commonly, infections (25). Recently COVID-19 (coronavirus disease) vaccinations were also reported as triggering factors in some case reports (26–30).

The key concept in the pathogenesis is the priming of the neutrophil granulocytes. During priming, neutrophil responsiveness is amplified by an activating stimulus by prior exposure to an agent. Examples of priming agents include tumor necrosis alpha (TNFα), interleukin (IL)-1,−6, interferon-gamma (IFNγ), lipopolysaccharides, substance P, and granulocyte-macrophage colony-stimulating factor (GM-CSF) (31, 32). Additionally, complement factor 5a (C5a) from the alternative complement pathway can also prime neutrophils by binding its receptors (32, 33). As a consequence of priming, antimicrobial activity increases, and MPO or PR3 antigens are expressed on the cell membrane. Cross-binding of the antigens and the surface Fcγ receptors by ANCA causes uncontrolled activation, resulting in excessive formation of cytokines, reactive oxygen species (ROS), lytic enzymes, and neutrophil extracellular traps (NETs) (31, 33). NETs are mesh-like structures of nuclear and mitochondrial decondensed chromatin, histones, cytoplasmic, and granule proteins (34). By exposing intracellular molecules, granular enzymes (e.g., PR3 and MPO), and ROS, NETs play a role in both vascular damages by a direct cytotoxic effect on endothelial cells and the generation of MPO-, PR3-, and atypical ANCA antibodies (34, 35).

MPO rests in the azurophil granules, bound to neutrophil elastase (NE), cathepsin G, lactoferrin, PR3, and lysozyme, forming azurosomes. MPO aids the release of neutrophil elastase into the cytoplasm by oxidizing it. NE binds to F-actin and degrades the cytoskeleton, making its way to the nucleus. Cytoskeletal degradation inhibits concomitant phagocytosis, committing cells to NETosis. NE also cooperates with gasdermin D in the destruction of the nuclear envelope by forming pores and allowing water influx. Entering the nucleus, NE and MPO decondense chromatin by degrading histones (36). This process is also promoted by the peptidyl arginine deiminase type 4 (PADI4) enzyme, which catalyzes histone arginine conversion to citrulline, reducing positive charge and loosening the compact chromatin structure (34, 35). Another detrimental effect of NETs is thrombocyte aggregation and enhancing coagulation (37). NETs serve as a scaffold in which platelets and red blood cells can accumulate, while H3 and H4 histones induce platelet aggregation. Furthermore, extranuclear DNA and histones serve as danger-associated molecular patterns (DAMPs) and induce apoptosis, necrosis, necroptosis, and pyroptosis leading to further inflammation (34). NET formation normally is counterbalanced by the activity of the endonuclease enzymes, e.g., Deoxyribonuclease-1 (DNASE1). Excessive formation and/or impaired clearance have a pathogenic role in autoimmune diseases, e.g., AAV, and systemic lupus erythematosus (38).

NET antigens are presented to naive T cells by dendritic cells that differentiate into CD4+ T helper (Th) cells. CD4+ T cells promote the differentiation of B-cells into ANCA-producing plasma cells. Cell-free DNA stimulates B-cells *via* Toll-like receptor 9 (TLR9), enhancing antibody production (33). Activated neutrophils release B cell-activating factor (BAFF) or B lymphocyte stimulator (BLyS) which is involved in enhancing B cell receptor signaling by upregulating the expression of the co-receptors CD21 and CD19, and prolonging B-cell survival by decreasing pro-apoptotic proteins (39).

Necrotizing granulomas are structures which can serve as a niche to induce differentiation and maturation of T cells. Macrophage-T cell interaction is the *sine qua non* of granuloma formation. In the early phase of granuloma formation, neutrophil invasion and the release of lytic enzymes result in the formation of a necrotic core lesion. Over time, macrophages are recruited, primarily to phagocytose the apoptotic debris. This process is called efferocytosis. A defect in efferocytosis is postulated to be a key step in the formation and in the inability of resolution of the granulomas. Anti-PR3 antibodies halt the clearance of PR3-expressing or ANCA opsonized cells and increase pro-inflammatory cytokine and chemokine release, attracting further neutrophils and T-cells (40). IL-23 tends to polarize T cells toward the TH17 phenotype (33). This subset of T cells releases a great quantity of IL-17,−21,−22, and TNFα, which leads to a vicious circle by stimulating macrophages and priming neutrophils. IL-17 is also implicated to have a major role in granuloma formation by activating endothelial and epithelial cells, causing macrophage and dendritic cell fusion (41).

To conclude, neutrophils play a key part in tissue damage in AAV, but B- and T-lymphocytes have also an important role to maintain the pathognomonic state, providing a wide range of targets for therapeutic intervention.

## Diagnosis of ANCA-Associated Glomerulonephritis

### Symptoms

Renal involvement in AAV is heterogeneous. The most aggressive renal manifestation of AAV is the rapidly progressive glomerulonephritis syndrome (RPGN), with a marked decrease in glomerular filtration rate (GFR) over some weeks or months, sometimes accompanied by oliguria (2). AAV, however, frequently appears as persistent microscopic and dysmorphic hematuria, sub-nephrotic proteinuria, and systemic hypertension, with or without azotemia (2, 42). Such clinical pictures mandate the serological investigation of ANCAs, and according to the latest guidelines, a solid ANCA-positivity with relevant clinical symptoms may be sufficient to start the remission induction treatment without renal biopsy (43). However, the biopsy is highly recommended and provides the “gold-standard” diagnostic results, especially in the case of an ambiguous serology or unusual clinical presentation.

**Figure 1:**
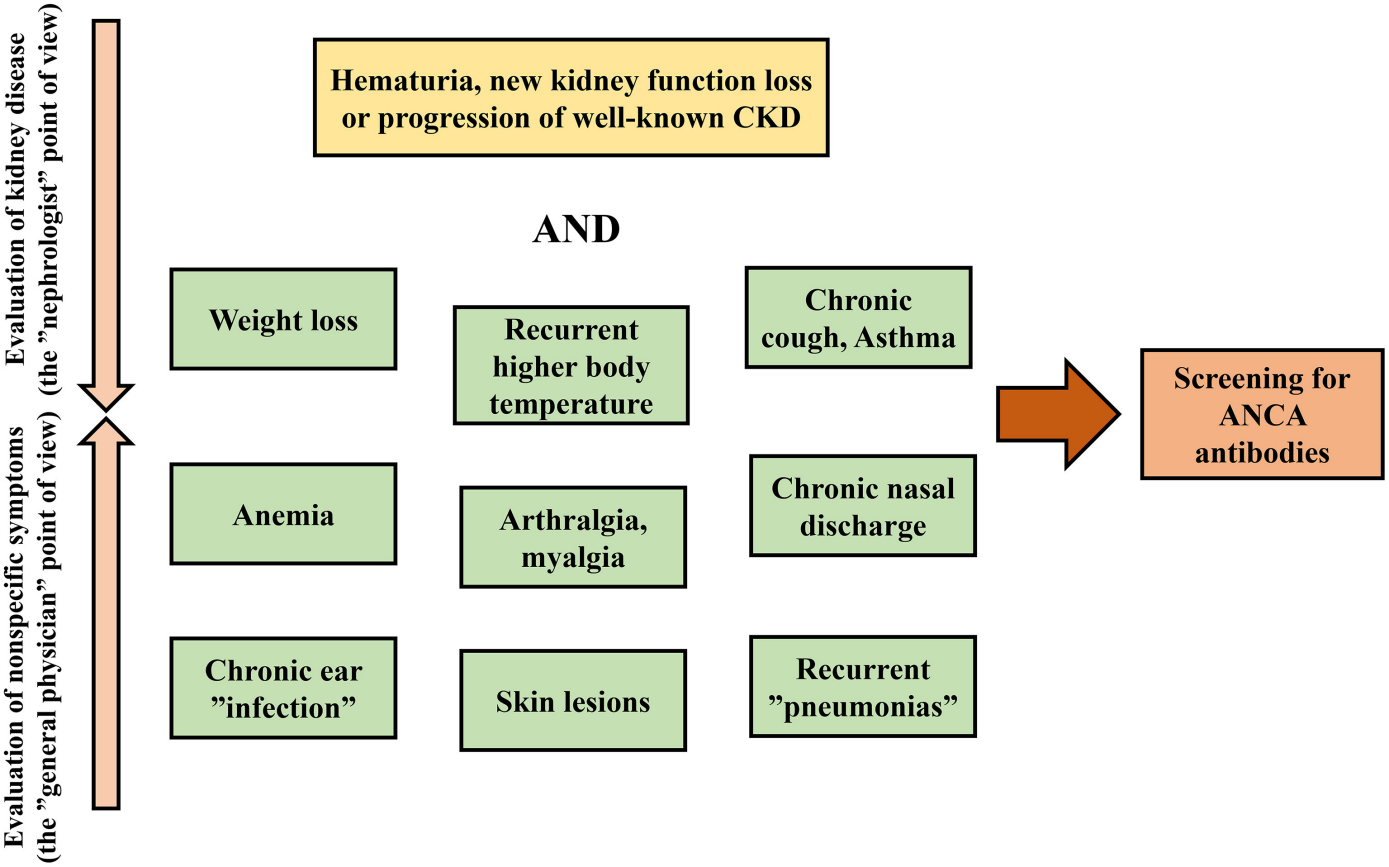
First steps of the diagnostic approach to ANCA-associated glomerulonephritis.

Considering the systemic nature of the disease, the involvement of various organs may complete the clinical picture, and the exclusion of alternative diagnoses, especially infections, is a crucial aspect of the evaluation. Extra-renal manifestations are also particularly important clues for the prompt diagnosis of ANCA-associated glomerulonephritis when rapid renal involvement is not present (Table 1) (2, 42, 44–74). Fever, malaise, anorexia, weight loss, arthralgia, and myalgia are common nonspecific manifestations of autoimmune diseases that should always raise the possibility of systemic vasculitis. Most of the key symptoms such as chronic nasal discharge, nasal polyps, sinusitis, recurrent otitis, persistent cough, skin lesions, etc. (Figure 1) may be mild and the patients may not attribute importance to them, resulting in diagnostic delay. Misdiagnosis may also be due to the presence of rare AAV-associated symptoms. For example, gastrointestinal manifestation is thought to be a rare complication of AAV, however, several case reports support the opposite. Even with histological evaluation, the diagnosis can be difficult, as the granulomatous lesions of GPA may make the involvement almost indistinguishable from inflammatory bowel disease (57, 69).

**Table 1:** Renal and the most common extra-renal symptoms of ANCA-associated vasculitis.

	**AAV**		
	**MPA**	**GPA**	**EGPA**	**RLV**
**Serology**	50% p-ANCA, 40% c-ANCA, 10% negative	70% c-ANCA, 25% p-ANCA, 5% negative	40% p-ANCA, 5% c-ANCA, 55% negative	70% p-ANCA, 20% c-ANCA, 10% negative
Constitutional symptoms	Fever, malaise, anorexia, weight loss, arthralgia, myalgia, flu-like symptoms.			
Pathology	Necrotizing vasculitis with few or no immunocomplexes. Small to medium vessels are affected: capillaries, venules, arterioles.			
		Granulomatous vasculitis	Granulomatous eosinophil-rich vasculitis Eosinophilia	
Renal involvement	90%	80–90%	15–20%	100%
	Hematuria, proteinuria, renal failure, rapidly progressive glomerulonephritis, hypertension.			
			Ureteral stenosis	
Cutaneous manifestations	40%	20%	60%	NA
	Palpable purpura, petechiae, ecchymosis, livedo reticularis, livedo racemose, subcutaneous nodules.			
		Pyoderma gangrenosum, ulcerated-centered and crusted nodules	Hemorrhagic vesicles and bullae, erythematous papules or plaques, erythema-multiforme-like lesion, ulcerated-centered and crusted nodules	
Ear-nose-throat	10–30% nasal congestion, sinusitis, sensorineural hearing loss.	60–85% nasal congestion, persistent rhinorrhea, recurring epistaxis, hyposmia, anosmia, sinusitis with purulent or bloody nasal discharge, nasal septum perforation, saddle-shaped deformity, otitis media, conductive and sensorineural hearing loss, mastoiditis, otitis externa, mucosal and palatal ulcers, palatal perforation, strawberry-like hyperplastic gingivitis, subglottic stenosis – voice changes, stridor, cough, shortness of breath, paratracheal pseudotumor	50–70% allergic rhinitis, chronic rhinosinusitis, bilateral nasal polyps, nasal crusting, granulomatous otitis media, hearing loss	NA
Pulmonary involvement	10–30% diffuse pulmonary hemorrhage, 45% interstitial lung disease, pulmonary consolidation, pleuritis 7% necrotizing granulomatous inflammation (lung nodules), 5% asthma 50% tracheal-bronchial disease.	5–45% diffuse pulmonary hemorrhage, 25% interstitial lung disease, pulmonary consolidation, pleuritis 50% necrotizing granulomatous inflammation (lung nodules), 10% asthma, 15–55% tracheal-bronchial disease, bronchiectasis	5% diffuse pulmonary hemorrhage, 15% necrotizing granulomatous inflammation (lung nodules), 70–100% asthma Pleuritis	NA
Gastrointestinal involvement	35–60% abdominal pain, nausea <5% Persistent diarrhea, bloody stool, ischemia	<5% abdominal pain, nausea, Persistent diarrhea, bloody stool, ischemia.	20% gastrointestinal erosion, pain, motility disorders, eosinophilic gastroenteritis	NA
Nervous system involvement	70% polyneuropathy, mononeuritis multiplex, 5–10% cranial neuropathy, 5% cerebral vasculitis <5% cerebral vasculitis, Posterior reversible encephalopathy, Hypertrophic pachymeningitis	10–45% polyneuropathy, mononeuritis multiplex, 10–45% pseudotumor orbitae 5–10% cranial neuropathy, 5% cerebral vasculitis <5% meningitis, Posterior reversible encephalopathy, hypertrophic pachymeningitis, pituitary gland and stalk involvement	75% peripheral neuropathy, mononeuritis multiplex, Subarachnoid and cerebral hemorrhage, cerebral infarction, cranial nerve palsy	NA
Ophthalmologic manifestations	Scleritis, episcleritis, conjunctivitis, corneal ulceration, retinal vasculitis, retinal detachment, vitreous hemorrhage.			NA
		Ophthalmoplegia, proptosis, chemosis, orbital pain		
Cardiovascular involvement	30%	30%	15–50%	NA
	Pericarditis, myocarditis, conduction system abnormalities, valvular involvement, venous thromboembolism, atherosclerosis.			

*MPA, microscopic polyangiitis; GPA, granulomatosis with polyangiitis; EGPA, eosinophilic granulomatosis with polyangiitis; RLV, renal limited vasculitis; ANCA, anti-neutrophil cytoplasmic antibody; p, perinuclear; c, cytoplasmic; NA, not applicable*.

In summary, considerable heterogeneity in symptoms and severity across the patients hinder the diagnosis and may delay proper treatment of the disease. The diagnosis of AAV starts with thinking of it.

### Classification Criteria

Since symptoms of vasculitis can be excessively overlapping, the Diagnostic and Classification Criteria in Vasculitis (DCVAS) study developed classification criteria to differentiate them. Results were published in 2022. These revised classification criteria were needed to increase sensitivity compared to the 1990 American College of Rheumatology (ACR) criteria. Since 1990, ANCA testing became widely available, as well as diagnostic imaging tools. On top of it, the recent classification included patients with MPA, which was not the case with the previous one. Another advantage is the shifting toward weighted criteria which enables a more discriminative classification (75–77).

### Laboratory Findings

The diagnostic hallmarks of AAV are the anti-neutrophil antibodies. Their diagnostics are based on indirect immunofluorescence (IIF) of ethanol-fixed neutrophils or enzyme-linked immunosorbent assay (ELISA). IIF is more sensitive, while ELISA is more specific. Their use varies around the world and between laboratories. Based on IIF, there are two types of fluorescence patterns with ANCA positive sera: cytoplasmic (c-ANCA), and perinuclear (p-ANCA). The mixture of these antibodies is defined as atypical ANCA. Antigens are located in the granules. Cytoplasmic ANCA pattern can be detected throughout the cytoplasm, and it is most commonly directed against proteinase 3 (PR3). The perinuclear pattern is artifactual and results from alcohol fixation. After fixation, positively charged granular constitutes, especially myeloperoxidase (MPO), rearrange to the negatively charged nucleus membrane and can be stained with anti-myeloperoxidase antibodies (44, 78).

ANCA antibodies are positive in 80–95% of ANCA-associated vasculitis patients. PR3- is strongly linked to GPA, while MPO-ANCA is to MPA and EGPA. Renal limited disease is usually MPO-ANCA positive as well (79). Nonetheless, 20–30% of the patients with clinical AAV have atypical ANCA, and 10–20% of them are ANCA negative, posing a diagnostic challenge and potential treatment delay (80–82). On the other hand, naturally occurring ANCA antibodies can also be detected in healthy individuals, accounting for false-positive cases.

Atypical ANCA antibodies can be detected in drug-induced vasculitis, inflammatory bowel diseases, rheumatoid arthritis, and systemic lupus erythematosus (83). Perinuclear-ANCA positivity is detected in inflammatory bowel diseases, especially in ulcerative colitis and in autoimmune hepatobiliary diseases (84). ANCA positivity may be noticed in bacterial endocarditis, chronic infections, malignancy, myeloma multiplex, cryoglobulinemia, idiopathic pulmonary fibrosis, and cystic fibrosis (81, 85–89). IgA (immunoglobulin A) nephropathy cases with ANCA positivity were also reported (35, 90). Anti-GBM (glomerular basal membrane) disease may also present with ANCA positivity, and in 5% of the cases, ANCA antibodies precede the development of anti-GBM antibodies by months (91–93). On the other hand, ANCA positivity (usually MPO-ANCA) in anti-GBM patients occurs in 35% of patients. Anti-GBM positive AAV patients' disease resembles more that of anti-GBM disease, and they have a higher risk of end-stage renal disease (ESRD) (19, 94).

In terms of complement levels, there is a controversy in the literature. Some authors declare that complement levels stay within the normal range, while others state that complement levels rise significantly in AAV, as a consequence of fulminant immune system activation (32, 95). Moreover, low levels of complement are also reported. The decreased level of C3 is associated with worse renal outcome which emphasizes the activation and the possible pathognomic role of the alternative pathway (96, 97).

Thrombocytosis, normochromic normocytic anemia, and leukocytosis are common findings in complete blood count. EGPA manifests with eosinophilia, which is commonly >10% of the total leukocyte count and is frequently accompanied by elevated serum IgE levels. Although C-reactive protein (CRP) and erythrocyte sedimentation rate elevate, these markers have low specificity (98).

Renal manifestation is evaluated by serum creatinine, blood urea nitrogen, and urine analysis. Proteinuria, hematuria, and urine sediment finding with dysmorphic erythrocytes and red blood cell casts aid the prompt diagnosis of the glomerular involvement.

### Kidney Biopsy

Since pathological features in the specimen do not alter the choice and the duration of the therapy currently, the need for renal biopsy in all AAV patients is controversial. The EULAR/ERA-EDTA (European League Against Rheumatism/European Renal Association) recommendations in 2016 supported the need for a renal biopsy to assist the diagnosis and prognosis (Level of evidence 3; grade of recommendation C) (99). In selected cases, where the clinical presentation is compatible with ANCA-associated vasculitis and ANCA positivity is solid, and there is a low probability of secondary vasculitis and false-positive ANCA, treatment can be commenced without renal biopsy (81). Out of consideration of prognostic factors, however, sampling can be performed (100). In case of an ambiguous serology result (negativity or positivity in low titer, atypical antibodies) or unusual clinical presentation, the biopsy is highly recommended. In these cases, the principal role of the biopsy is to provide diagnostic information. The diagnostic yield of a kidney biopsy is especially high in GPA (91.5%) (101). According to the newest KDIGO (Kidney Disease Improving Global Outcome) 2021 guideline on glomerular diseases, renal biopsy remains the gold standard in the diagnostic process, therefore, it should always be considered in patients with ANCA-associated glomerulonephritis. On the other hand, when the clinical presentation is unquestionable, waiting for a biopsy should not delay the start of the proper treatment and biopsy can be performed later, “when feasible.” This phrase, however, may leave an alternative to skip biopsy in some patients, especially in the elderly, and in those, who are in poor general condition with obvious clinical signs for AAV and with good response to the remission induction therapy (It is important to note, that the statements regarding the need of a kidney biopsy were a practice point, not a recommendation, because of the lack of conclusive evidence.) (43).

The histopathological feature of ANCA-associated glomerulonephritis is pauci-immune small-vessel vasculitis. The term “pauci” refers to the scarcity of immunoglobulin and complement deposition detected by indirect immunofluorescence. In the earliest phase, light microscopy reveals neutrophil invasion occurring in the vessel walls, and it is gradually overtaken by mononuclear leukocytes. Leukocyte fragmentation (leukocytoclasia) can be observed.

**Figure 2:**
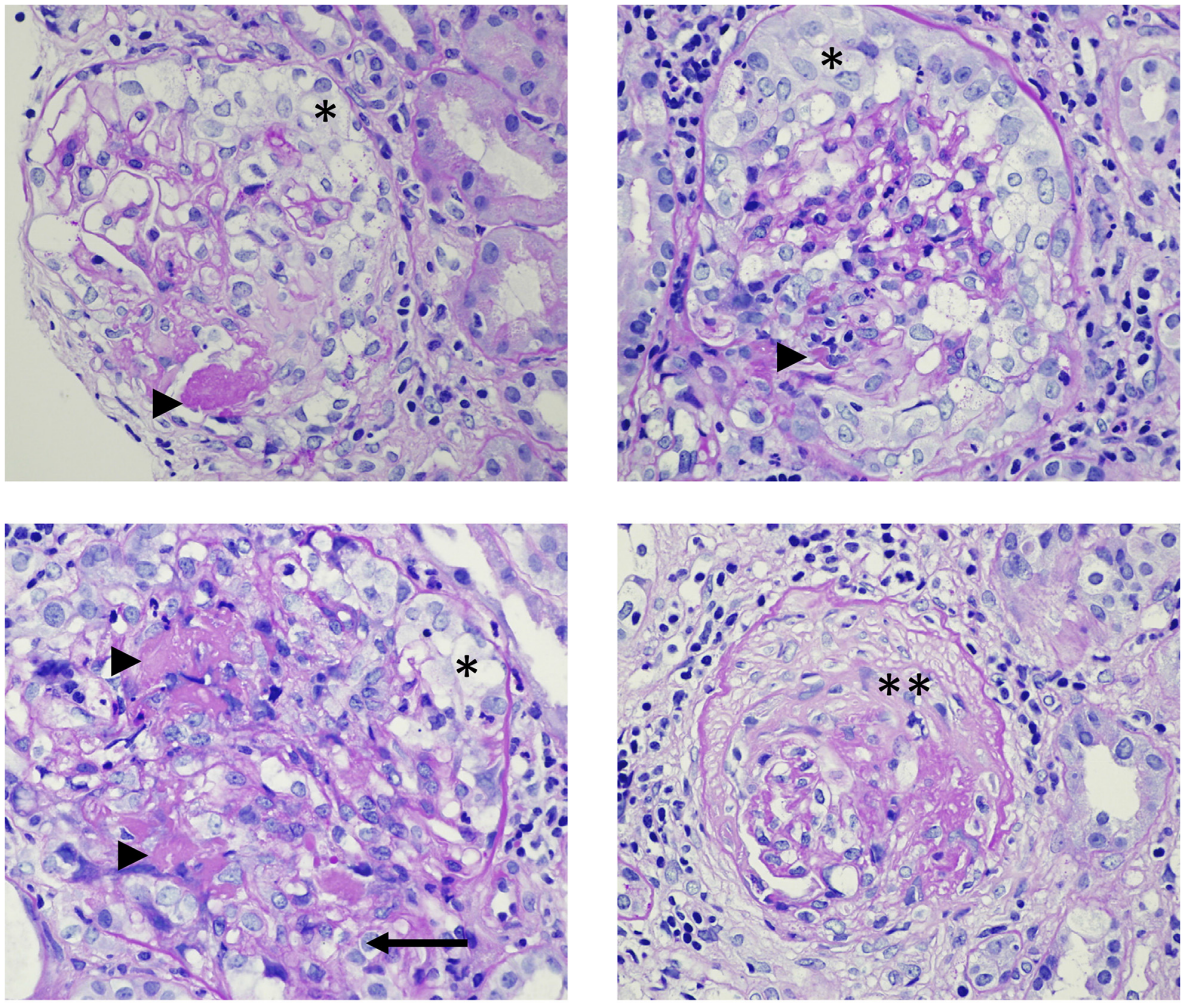
Histopathology images from the renal biopsy of a patient with ANCA-associated glomerulonephritis. The patient has renal limited vasculitis with anti-myeloperoxidase positivity, causing rapidly progressive glomerulonephritis syndrome. Arrowheads: fibrin; *: cellular crescents; **: sclerotic crescent; arrow: rupture of the Bowman's capsule. Light microscopy, Periodic Acid-Schiff (PAS) staining, magnification: 600x.

Segmental fibrinoid necrosis, with or without cellular and fibrocellular extracapillary crescents are common findings in early vascular lesions (Figure 2). Although severe acute lesions may appear as large concentric crescents or widespread sclerosis it is usually a hallmark of chronic inflammation (102). Apart from glomerulonephritis, AAV patients might have renal arteritis. On rare occasions, medullary angiitis manifests as medullary interstitial disease and papillary necrosis in severe cases (103). There are main histopathologic types (Berden classification) that provide information about the prognosis: sclerotic (>50% globally sclerotic glomeruli), focal class (>50% normal glomeruli), crescentic class (>50% of glomeruli with cellular crescents), or mixed class in which no dominant feature can be detected. Renal survival (time to end-stage renal failure) at 5 years from the time of the diagnosis was 93% in patients with focal, 76% with crescentic, 61% with mixed, and 50% with sclerotic categories at the time of the diagnosis (100).

GPA and EGPA patients rarely have necrotizing granulomatous lesions in the kidney biopsy specimens. Concomitant eosinophilic infiltration may be detected in GPA and it is prominent in EGPA (104).

Repeat biopsies to monitor kidney function and the success of therapy are not performed routinely, however, they may have a role in assessing disease activity. Clinical biomarkers, such as hematuria and serum creatinine do not reflect disease activity specifically and sensitively enough, thus they can be mistaken for relapses. Approximately half of the patients who underwent rebiopsy had alteration in the Berden classification, up-or downstepping in the classification hierarchy (105, 106). This finding suggests that interval biopsy could aid clinicians to develop more appropriate and personalized treatment and predict kidney outcomes more precisely than the initial biopsy.

### Other Biopsy Sites

As discussed above, cutaneous manifestations can appear in a wide variability. Thus, diagnosis can be challenging, in which skin biopsy could aid the work. Lung biopsy is rare and only conducted in the absence of systemic manifestations or when serology cannot prove the diagnosis. It is to rule out alternative diagnoses since granulomas do not have a specific sign on imaging examinations, they can mimic tumors and infections as well (72).

## Disease Activity

The chronic relapsing-remitting nature of AAV necessitates tight follow-up of disease activity to provide appropriate management. The Birmingham Vasculitis Activity Score V3.0 (BVAS) is an approved, easily available, and effective tool to evaluate disease severity in everyday clinical work. The scoring system scrutinizes 9 organ involvement, and 66 attributes altogether and assesses the symptoms according to their persistent or intermittent nature. Clinical features are weighted according to their clinical relevance (107, 108). The score has a prognostic value in the short and medium-term mortality and also suggests responsiveness to therapy (108). Other scores, such as Vasculitis Damage Index (109), Disease Extent Index (110), Five Factor Score for EGPA (111) are also validated and reliable (112).

## Treatment

One of the main aims of this review is to summarize the recent recommendations for the management of ANCA-associated glomerulonephritis in light of two recent guidelines: KDIGO and the American College of Rheumatology/Vasculitis Foundation (ACR) guideline from 2021 (43, 113). As glomerulonephritis is more prominent in GPA/MPA, EGPA treatment is not discussed in this section.

### Induction of Remission

**Figure 3:**
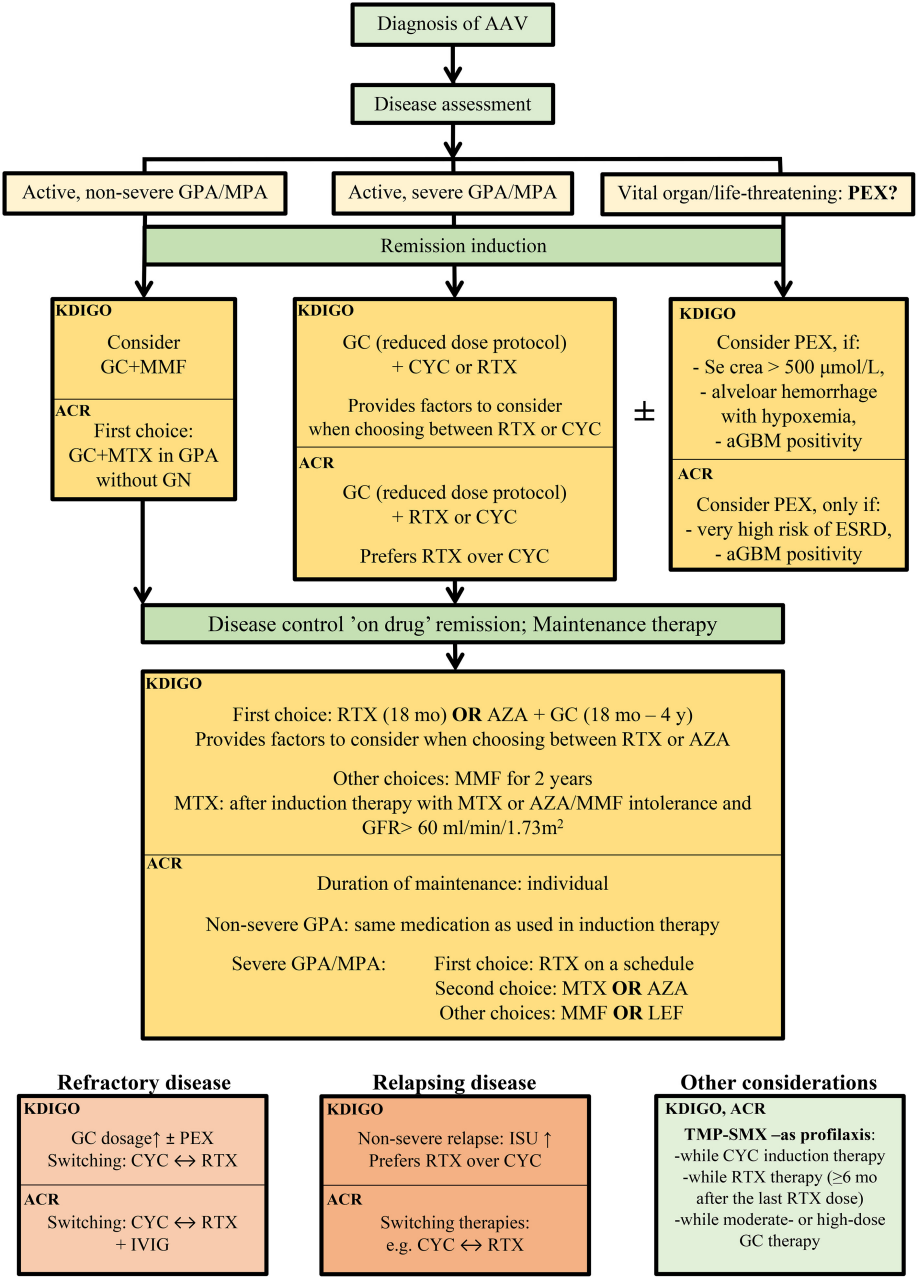
Therapeutic recommendation of the remission induction therapy in ANCA-associated glomerulonephritis, according the current guidelines, KDIGO 2021 Clinical Practice Guideline for the Management of Glomerular Diseases (43) and American College of Rheumatology 2021 (113). AAV, ANCA-associated vasculitis; KDIGO, Kidney Disease Improving Global Outcome; ACR, American College of Rheumatology; aGBM, anti-glomerular basal membrane antibody; AZA, azathioprine; crea, creatinine; CYC; cyclophosphamide; ESRD; end-stage renal disease; GC, glucocorticoids; GFR, glomerular filtration rate; GN, glomerulonephritis; GPA, granulomatosis with polyangiitis; ISU, immunosuppressive therapy; IVIG, intravenous immunoglobulin; LEF, leflunomide; MMF, mycophenolate mofetil; mo, months; MPA, microscopic polyangiitis; MTX; methotrexate; PEX; plasma exchange; RTX, rituximab; TMP-SMX, trimethoprim/sulfamethoxazole; y, years.

The treatment of AAV varies according to organ involvement and disease severity. ANCA-associated glomerulonephritis is considered an organ- or life-threatening disease, therefore, demands induction and maintenance immunosuppression (Figure 3).

The standard induction immunosuppression therapy is glucocorticoids combined with cyclophosphamide (CYC) and/or rituximab (RTX) (Figure 3, Table 2).

**Table 2:** Remission induction therapy in ANCA-associated glomerulonephritis according to the KDIGO 2021 Clinical Practice Guideline for the Management of Glomerular Diseases (43).

**Corticosteroid**	**Iv. corticosteroid**	**Oral corticosteroid**
	7–15 mg/kg methylprednisolone (max. 1 g/day) for 3 days, followed by oral corticosteroid, and tapered in 3–6 months	1 mg/kg/day prednisolone (or its equivalent) (max. 80 mg/day), tapered in 3–6 months
**Immuno-suppressive** **drug dosing**	**Oral CYC**	**Iv. CYC**	**RTX**	RTX and iv. CYC*	**MMF**
	2 mg/kg/day for 3–6 months	Six pulses of 0.5 mg/m^2^ or 15 mg/kg (max. 1.2 g) at weeks 0, 2, 4, 7, 10, 13.	1 g at weeks 0 and 2 OR 375 mg/m^2^ weekly for 4 weeks	RTX 375 mg/m^2^/week for 4 weeks plus 15 mg/kg CYC at weeks 0 and 2 OR	2,000 mg/day (divided doses), may be increased to 3,000 mg/day
	Reduction for age: 60 y, 1.5 mg/kg/day 70 y, 1 mg/kg/day Reduce by 0.5 mg/kg/day for GFR <30 ml/min/1.73 m^2^	Reduction for age: 60 y, 12.5 mg/kg 70 y, 10 mg/kg Reduce by 2.5 mg/kg for GFR <30 ml/min/1.73 m^2^		RTX 1 g at weeks 0 and 2 plus six pulses of 500 mg CYC fortnightly	

CYC and RTX combination therapy (*) is not recommended by the American Collaged of Rheumatology 2021 guideline (113).

*IV., intravenous; CYC, cyclophosphamide; RTX, rituximab; MMF, mycophenolate mofetil, y, years*.

Glucocorticoid (GC) treatment may include high dose (1–3 g) of intravenous (IV) methylprednisolone in patients presenting with RPGN, and daily oral glucocorticoids tapered over 5 months in all patients with organ-threatening disease. IV methylprednisolone is routinely administered in severe diseases; however, it has never been tested in randomized controlled trials. The recent ACR guideline recommends - as an ungraded position statement - either IV pulse or high-dose oral glucocorticoid treatment as a part of the initial therapy of active, severe GPA/MPA (113). As prolonged administration and high dose oral corticosteroids are associated with high risk of adverse events, recent recommendations favor therapies with accelerated taper, thus lower glucocorticoid requirements. According to the results of the Plasma Exchange and Glucocorticoids for Treatment of Anti-Neutrophil Cytoplasm Antibody (ANCA)-Associated Vasculitis (PEXIVAS) trial, rapid tapering of glucocorticoids may eliminate some side effects without altering the efficacy of the therapy (114). Based on the protocol and results of the PEXIVAS study, both KDIGO and ACR 2021 guidelines recommend the reduced-dose glucocorticoid regimen over the standard dose in patients with active, severe GPA/MPA (Level of evidence: very low to moderate) (43, 113). The use of rituximab in combination with intravenous or oral cyclophosphamide permits lower glucocorticoid exposure as well (115). The oral complement C5a receptor inhibitor, avacopan as a potential alternative to glucocorticoids, may also reduce glucocorticoid-related toxicity and improve patent quality of life (116).

Cyclophosphamide has been used for induction immunosuppression of AAV for almost 50 years now (117). The European Vasculitis Study Group compared daily oral CYC with intravenous pulse CYC regimens (CYCLOPS trial) and found that both routes of admission induced remission. As a result of the decreased cumulative dose, pulse CYC was associated with fewer cases of leukopenia and infection, but also demonstrated a tendency toward a higher risk of disease relapse (118). Older adults and patients with impaired renal function require dose reductions (Table 2) (43).

The Rituximab for ANCA-Associated Vasculitis (RAVE) and the Rituximab versus Cyclophosphamide in ANCA-associated Renal Vasculitis (RITUXVAS) trials compared RTX- to CYC-based induction treatment. Remission rates, relapse rates, or adverse events (119, 120) were similar for the two drugs. RTX is also highly efficient in relapsing disease to re-induce remission. (121). RTX provided a higher initial remission rate in aPR3-ANCA positive patients (122). Phenotypic characterization of B cells is suggested to aid the selection of therapy. AAV patients with a higher level of class-switched memory B cells or IgD^−^CD27^−^ B cells, rituximab is likely to be more effective than cyclophosphamide, enabling earlier glucocorticoid tapering and better survival rates (123). In severe kidney disease (serum creatinine > 4 mg/dL or 354 μmol/L), however, no randomized clinical trials were conducted with RTX alone, only in combination with CYC (124). The potential superiority of the combination therapy is currently under investigation in the ongoing ENDURRANCE-1 randomized-controlled trial (NCT03942887). The main difference between the recent ACR and KDIGO guidelines from 2021 is related to the CYC vs. RTX use in the remission induction therapy. ACR recommends RTX over CYC for patients with active, severe GPA/MPA (113), while the KDIGO recommendation does not declare superiority but provides factors to consider while choosing from the protocols. RTX is preferred in children, adolescents, and reproductive-aged patients, in frail older adults, in relapsing or PR3-ANCA disease, or when glucocorticoid-sparing is especially important. CYC remained the first choice in severe kidney disease (serum creatinine > 4 mg/dL or 354 μmol/L), because of the lack of evidence from randomized controlled trials (43). Based on the protocol of the RITUXIVAS study, KDIGO provides a combination regimen of RTX and CYC (practice point, not a recommendation), while ACR does not suggest combined therapy.

A randomized controlled trial compared mycophenolate mofetil (MMF) to pulse cyclophosphamide and found mycophenolate non-inferior for remission induction in patients with estimated GFR >15 ml/min/1.73 m^2^. However, relapse rates were higher, mostly in PR3-ANCA positive patients (125). This suggests that MMF could be utilized as first-line induction therapy in MPO-ANCA positive individuals with moderate renal damage (126). In fact, the latest KDIGO recommendation also included MMF as induction in the therapeutic palette (Table 2) (43). According to the ACR guideline, MMF may be a secondary choice in active, non-severe GPA, or tertiary choice in the maintenance therapy of active severe GPA/MPA (113).

Methotrexate (MTX) is the first choice combined with GC in active, non-severe GPA according to the ACR guideline (113). MTX is recommended over CYC or RTX in this patient group, based on its less toxicity and greater clinical experience. The definition of non-severe disease is vasculitis without organ- or life-threatening manifestations (e.g., rhinosinusitis, mild systemic symptoms, mild arthritis, etc.), so it is not to be considered in active glomerulonephritis. The KDIGO group focused on AAV with glomerulonephritis and recommends MTX only as maintenance therapy if azathioprine (AZA) or MMF are not tolerated and eGFR >60 ml/min/1.73 m^2^.

In refractory diseases, glucocorticoid dosage may be increased with the addition of CYC (if previously RTX was used) or with the addition of RTX (if previously CYC was used). Plasmapheresis may also be part of the treatment in poor responders (43), according to the KDIGO guideline. ACR recommends switching between CYC or RTX therapies in active, severe refractory GPA/MPA over combining the two therapies. Intravenous immunoglobulin (IVIG) may be also added to refractory GPA/MPA (ACR recommendation) (113). Secondary causes of AAV should always be excluded in the case of refractory disease (drug- or malignancy-induced AAV, infections). The importance of the kidney biopsy is clear in refractory diseases to rule out other underlying kidney diseases and evaluate disease activity or chronic, irreversible tissue damage.

### Plasmapheresis

Since anti-neutrophil-associated antibodies are pathogenic in AAV, their rapid removal by plasmapheresis may control disease activity and prevent organ damage. Plasma exchange also removes cytokines and inflammatory mediators that enhance the harmful effects of the autoantibodies (127). The addition of plasmapheresis to standard therapy, however, is a controversial issue. The Plasma Exchange for Renal Vasculitis (MEPEX) trial demonstrated that plasmapheresis improved renal outcome in patients with severe kidney disease (128). In the PEXIVAS trial, however, plasmapheresis conferred survival benefits neither in patients with eGFR <50 ml/min/1,73m^2^ nor in patients with lung hemorrhage (114), and it only reduces the risk of ESRD within 12 months. On the other hand, it increases the risk of serious infections, particularly in the first year of treatment (129). The lack of kidney biopsy during the enrollment in this trial, however, limits the generalizability of the results. According to the American Society of Apheresis (ASFA), plasmapheresis is recommended in cases of biopsy-proven RPGN, signs of active inflammation in the glomeruli such as fibrinoid necrosis or crescents with a small degree of fibrosis, and a fulminant clinical course (130). This recommendation overlaps with the latest KDIGO guideline (43), where plasmapheresis is recommended only in the case of severe RPGN (serum creatinine > 500 μmol/L or 5.65 mg/dL), if alveolar hemorrhage with hypoxemia, or anti-GBM positivity is present (Practice point, not a recommendation). ACR guideline is also against the routine addition of plasma exchange in active severe GPA/MPA with glomerulonephritis (level of evidence is low to high), and plasmapheresis may be considered only in patients with a high risk for ESRD or with anti-GBM positivity (113) (Figure 3).

### Maintenance Therapy

After the induction therapy, a lower level of immune suppression is required to maintain the achieved remission and prevent relapse. The most common maintenance therapy comprises a reduced dose of glucocorticoids with azathioprine (AZA) or RTX (without GC) (Table 3).

**Table 3:** Immunosuppressive maintenance therapy in ANCA-associated glomerulonephritis according to the KDIGO 2021 Clinical Practice Guideline for the Management of Glomerular Diseases (43).

**Rituximab**	**Azathioprine**	**Mycophenolate mofetil**	**Methotrexate**
500 mg twice at complete remission, and 500 mg at months 6, 12, and 18 (MAINRITSAN) OR 1,000 mg after remission induction and at months 4, 8, and 16 after the first infusion (RITAZERAM)	1.5–2 mg/kg/day at complete remission until 1 year after diagnosis, tapering by 25 mg every 3 months OR Extended protocol: 1.5–2 mg/kg/day for 18–24 months, then decrease dose to 1 mg/kg/day until 4 years after the diagnosis, then decrease by 25 mg every 3 months Glucocorticoids should also be continued at 5–7.5 mg/day (prednisolone-equivalent) for 2 years and then slowly reduced by 1 mg every 2 months	2,000 mg/day (divided doses) at complete remission for 2 years	Up to 25 mg/week if eGFR > 60 ml/min/1.73 m^2^

*eGFR, estimated glomerular filtration rate*.

AZA proved to be a safe and efficient alternative to oral CYC for maintenance therapy in the Cyclophosphamide vs. Azathioprine for Early Remission Phase of Vasculitis (CYCAZAREM) trial. Severe adverse events and relapse rates did not differ between the groups, however, AZA treatment resulted in fewer cases of leukopenia (131).

According to the Maintenance of Remission using Rituximab in Systemic ANCA-associated Vasculitis (MAINRITSAN) trial, RTX-treated patients experience fewer major relapses than AZA-treated peers, and RTX was superior to AZA in PR3-ANCA positive patients (132). There are more types of protocols with different doses of RTX in the clinical trials and comparative trial has not been conducted yet (500 mg IV every 6 months – MAINRITSAN scheme, approved by FDA (U.S. Food and Drug Administration) vs 1,000 mg IV every 4 months – RITAZAREM scheme), so the optimal protocol is yet to be determined (132, 133). However, the efficient but lower dose of RTX might be a better choice to reduce side effects. The efficiency of RTX vs. AZA treatment in relapsing disease is under investigation in the Rituximab vs. Azathioprine as Therapy for Maintenance of Remission for Anti-neutrophil Cytoplasm Antibody-associated Vasculitis (RITAZAREM, NCT01697267) trial (133).

As compared to AZA, maintenance therapy with MMF resulted in the same level of adverse effects, proteinuria, glomerular filtration rate, and disease activity score, but a higher relapse rate (IMPROVE) (134). MTX, however, may serve as an alternative for AZA, at least in patients with preserved renal function (eGFR > 60 ml/min/1,73 m^2^). According to the WEGENT trial, MTX- and AZA-based maintenance therapies are equally effective in preventing relapse and provide similar long-term outcomes (135).

While high doses of glucocorticoids and associated toxicity is a relevant concern in induction therapy, it must be noted that the prolonged administration of low-dose glucocorticoids seems to contribute to efficient maintenance therapy and also prevents relapses (136). According to KDIGO and ACR recommendations, AZA treatment has to be combined with low-dose glucocorticoids, whereas RTX treatment is not. In the MAINRITSAN trial, however, RTX was applied together with low-dose prednisone treatment for at least 18 months, and the prolonged glucocorticoid treatment could have contributed to the unexpectedly low major relapse rate (132).

The recommendations regarding the first choice of maintenance therapy are slightly different according to the most recent guidelines. KDIGO recommends either RTX or AZA with low-dose GC for maintenance (level and quality of evidence 1C) while providing factors to consider choosing the best therapy for individual patients. RTX is preferred in relapsing disease, in PR3-ANCA disease, frail or older adults, when GC-sparing is important, or in AZA-allergy. AZA is preferred with hepatitis B exposure, low baseline immunoglobulin G (IgG < 300 mg/dl) level, or when RTX is not available (43). MMF provides an alternative to RTX or AZA, while MTX can be used if kidney function is preserved, according to the KDIGO group. On the other hand, ACR recommends RTX on a schedule-based protocol in the maintenance therapy of active, severe GPA/MPA (level of evidence: very low to moderate) over other therapies and suggests IVIG administration if IgG level is low during RTX treatment and recurrent infections are present (113). MTX, AZA, MMF, and leflunomide could be secondary or tertiary choice during maintenance. In active, non-severe GPA, the first choice of maintenance is the same medication as the remission was reached with and therapy switching is needed only in relapsing disease (113) (Figure 3).

The optimal duration of maintenance treatment is yet to be determined. Too early withdrawal of therapy increases the risk of relapse, while prolonged immunosuppression is associated with greater toxicity. The current KDIGO guideline suggests AZA to be administered for at least 18 months and therapy may extend to 4 years (43). RTX maintenance was studied up to 18 months. While individually tailored rituximab regimens – administration upon B-cell return or serologic relapse – are as effective as fixed-schedule ones in preventing relapse, based on clinical experience and the limited power of the related trial, ACR recommends scheduled RTX re-dosing during remission maintenance (Level of evidence very low to low) (113, 137).

### Relapse: Monitoring and Therapy

Although induction protocols are commonly effective in remission induction, maintenance regimens vary in efficacy. Depending on the treatment regimens, relapse rates range from 30 to 50% at 5 years (138).

The rise in the ANCA titer is suggested as an early sign of the impending relapse. Doubling of ANCA-level in the previous 3 months provides an 11-times higher chance for relapse in the next 1.5 years (139). Nevertheless, it is still debatable whether the ANCA persistence or the rise could predict the disease activity and should lead to an abrupt change of therapy (140, 141). In many cases, ANCA persistence and even elevation in ANCA-titers are not associated with worsening clinical features, though ANCA persistence is often linked to partial remission or subclinical appearance, and a “second hit” is required to trigger a manifest vasculitis. In addition, ANCA-titers have a modest correlation with disease activity (140).

The reason behind this observation is not entirely clear, but it might trace back to the pathogenicity of ANCA: epitope specificity and sialylation ratio which are thought to regulate ANCA reactivity (142, 143).

Identifying clinical features and/or biomarkers to predict an imminent relapse reliably would be essential in the chronic care and follow-up in AAV patients and it remains a key research priority. Anti-PR3 positivity, granulomatous disease, lower serum creatinine at presentation, nasal Staphylococcus aureus colonization, ear-nose-throat involvement, and prior relapses convey a higher relapse risk (144). Though BVAS (v3) is usually used in clinical trials to measure symptoms objectively, it can be an appropriate tool in everyday practice to evaluate the symptoms of AAV. The relapse of the disease can affect a previously not involved organ system (145), so a proper, structured checklist of the possible symptoms (persistent and new-onset) aids the physicians' work. Online BVAS (v3) calculators are available and make it easy to register the patients' disease activity score in their documentation during the follow-up period (146). Both recent guidelines agree on immunosuppressive therapy dosing should not be based on ANCA titers only but the patient's clinical symptoms parallel with diagnostic findings should be considered in treatment decisions (43, 113).

Patients with severe relapsing disease should be treated by reintroducing induction therapy (43, 113). According to the current guidelines, RTX is preferred to be administered during the relapse, as higher remission rates were found with RTX compared to CYC in relapsing disease in a *post hoc* analysis of the RAVE trial, especially in PR3-ANCA positive patients (122). For patients with severe relapse while receiving RTX for remission maintenance, switching therapy to CYC is a better choice than receiving RTX for remission re-induction, especially if the patient recently received RTX (113). If CYC has been chosen to induce remission in relapsing disease, it is very important to calculate the cumulative dosage, as the risk of malignancies increases significantly above the total of 36 g of CYC administered (147). In mild relapses, CYC should be avoided (43).

High-dose (IVIG) has immunomodulatory effects *via* neutralizing complements, inhibiting the maturation and function of dendritic cells, inflammatory cytokines, enhancing the expansion of regulating T-cells, and decreasing Th1 and Th17 cells. It also helps to neutralize autoantibodies (148). IVIG is proved to be a beneficial adjunctive therapy not only in refractory but also in relapsing AAV diseases by decreasing disease activity and having an acceptable safety profile (149). IVIG can be used as a salvage therapy in active GPA/MPA in special patient groups when other therapies are contraindicated (e.g., during pregnancy) (113).

To conclude, recognizing patients with a high risk of renal relapse would benefit clinical care and therapy optimization. Clinical relapse occurs mainly at the initiation of the maintenance therapies or during their tapering (140) which suggests that the timing to treat relapse and precise therapy regimes still need to be elucidated.

## Chronic Care of Patients With ANCA-Associated Glomerulonephritis

### Prognostic Factors

As discussed above, histologic findings - according to the Berden classification - correlate well with the prognosis. Focal pattern is associated with the best, while sclerotic histology is related to the poorest outcomes. Ambivalently, the ESRD-free survival rate is surprisingly high in the sclerotic group: it remains 50% until 5 years and only lowers at around 7 years, while in other categories, it worsens gradually (100). The sclerotic group of patients cannot be considered homogenous. Patients with an eGFR of more than 15 ml/min/1.73 m^2^ at baseline have a substantially higher long-term ESRD-free survival rate, and their renal survival rate is approximately the same as in patients with mixed or crescentic pattern. Sclerotic histology with eGFR <15 ml/min/1.73 m^2^ however, is proposed to have a poor prognosis (150). Glomerular crescents are correlated with a better outcome than sclerosis, suggesting that active inflammation may be suppressed or even treated with immunosuppressive therapy, while chronic lesions are most likely to be irreversible. Sclerosis and its extent is associated with advanced chronicity: the longer the time from the diagnosis, the worse the prognosis (151, 152). ANCA Renal Risk Score (ARRS) predicts renal outcome according to the percentage of normal glomeruli, tubular atrophy, interstitial fibrosis, and renal function at the time of the diagnosis (153). Chronicity Score by Mayo Clinic/Renal Pathology Society (MCCS) includes glomerulosclerosis, interstitial fibrosis, tubular atrophy, and arteriosclerosis. Higher MCCS grades are associated with decreased renal function and poor outcome (154), as well as treatment resistance (155).

As regards clinical parameters, older age, lower GFR, and MPO-ANCA positivity are associated with poorer renal outcome (151). Treatment resistance was also anticipated by anti-MPO positivity, female sex, and black race (156). Patients who underwent B cell depletion and have a low or decreasing number of CD5+ B cells after repopulation are more likely to have a shorter relapse time. Pulmonary hemorrhage correlated with overall poor outcome as well (157, 158).

### Other Aspects of AAV

#### Increased Cardiovascular Risk in AAV

Cardiovascular diseases are the most common cause of mortality in AAV after the first year (67). Autoimmune abnormalities are known to play a role in atherogenesis and the acceleration of atherosclerosis (68). As a result, hypertension, the risk of ischemic heart disease, therefore cardiomyopathy, myocardial infarction, transient ischemic attack, and stroke increase in AAV patients (67, 159). Venous thrombotic events are also frequently seen in association with AAV (66, 160). Serosal involvement is rare compared to other autoimmune diseases and occurs mainly in EGPA (70). Besides the damage to the small vessels and chronic systemic inflammatory state, prolonged glucocorticoid treatment can enhance cardiovascular morbidity in AAV patients. It is, therefore, prudent to evaluate the patients' traditional cardiovascular risk factors (smoking habits, dyslipidemia, hypertension, diabetes mellitus, obesity, physical inactivity), and address them during their chronic care.

#### Risks of Malignancies

AAV is associated with an increased risk of cancer development, and malignancies particularly affect the urinary tract and the skin. Hematologic malignancies are also more frequent (161). Chronic inflammation is known to be a risk factor in oncogenesis. By creating excessive oxidative stress, enhancing cell proliferation, angiogenesis, and cell invasion, inflammation favors tumor-microenvironment (162). Long-standing immune activation leads to excessive immune cell proliferation and a greater risk of acquired genetic aberrations, and resistance to apoptosis, therefore to a rise of a malignant clone (163).

Immune surveillance is the process, in which the immune system recognizes precancerous or cancerous cells and eliminates them. Autoimmune diseases mean immune dysregulation, hence impaired removal of the altered cells. Surveillance is damaged by immunosuppressive therapies as well (164), and in addition, many of them have a direct cytotoxic and carcinogenic effect (161, 165).

CYC is particularly associated with oncogenic features. Its metabolites, acrolein, and chloro-acetaldehyde, cause DNA breakage. They are excreted in the urine and get in direct contact with the urothelium. The accumulation of toxic metabolites in the bladder can cause bladder cancer (161), the incidence of which may be decreased by hyperhydration and by the use of sodium 2-mercaptoethanesulfonate (MESNA) (161). As mentioned above, high cumulative CYC dosage increases the risk of malignancies (147).

Of note, rituximab does not increase the risk of malignancy compared to the general population (166).

#### Risks of Infections

Vasculitis and infections are tightly linked. Bacterial infections can initiate ANCA production and infections are common complications of immunosuppressive therapy. They are reported in 20–76% of the cases of which three-quarter takes place during induction therapy (167, 168). In fact, infections remain the major cause of death (159, 167, 168). Renal involvement raises the risk of serious infections, and it is linked to worse outcome as well as older age, higher BVAS score, diabetes, and smoking (168). Pneumonia is the most common form of infection, followed by sepsis, urinary tract, and skin infections (168). Bacterial infections owe up to 80% of the infections, and Staphylococcus aureus, Acinetobacter baumanni, Escherichia coli, Klebsiella species, Enterococci, and Pseudomonas aeruginosa are frequent underlying pathogens. Viral infections are usually caused by Cytomegalovirus, Varicella-Zoster, Influenzae, and Herpes simplex virus, and they occur in 10–15% of the cases. Fungal and parasitic infections contribute to about 10% of the infections, of which Pneumocystis jiroveci, Aspergillus fumigatus, and Candida species, are the most common pathogens (168, 169). For the prevention of opportunistic infections, low-dose sulfamethoxazole/trimethoprim or pentamidine is recommended for the course of the cyclophosphamide treatment, and at least 6 months after rituximab induction therapy (43, 113). Prophylactic antiviral and antifungal agents could be considered in severely immunocompromised patients (170).

Hepatitis B and C reactivation can occur as a consequence of immunosuppressive therapy; therefore, screening is advised before treatment. Preemptive antiviral prophylaxis (lamivudine, entecavir, or tenofovir) should be prescribed if the patient has a history of hepatitis B or C infection (171). To avoid the flare-up of latent tuberculosis, QuantiFERON-TB Gold screening is also rational prior to the initiation of the immunosuppressive treatment, however, there is a high rate of indeterminate results in patients with systemic vasculitis (172).

A severe but very rare infectious complication of rituximab treatment is progressive multifocal leukoencephalopathy (PML), caused by the JC polyoma virus. JC virus infects commonly and persistently the urinary tract of adults. The PML-associated neurotropic JC virus strain contains distinctive mutations in the surface loops of the major capsid protein, leading to escape from antibody-mediated neutralization. PML used to be the rare complication of HIV (human immunodeficiency virus) infected patients, but it has become more frequently related to immunosuppressive treatments with monoclonal antibodies. The cumulative reporting rates of confirmed PML in HIV-negative patients were at about two per 100 000 patients in the period 1998 to 2007, then increased to 7 to 8 per 100 000, which included patients with hematologic and oncologic diseases. The higher number of registered cases is partly due to the better awareness of the disease (173).

Live attenuated vaccines are contraindicated during immunosuppressive therapies. Inactivated vaccines are recommended at least 2 weeks prior to the initiation of the treatment. In case of rituximab, vaccines should be given at least 5 months after the last dose and at least 4 weeks before the administration (174).

### Renal Replacement Therapy in AAV

Despite the evolution of immunosuppressive therapy in AAV, 20–25% of the patients develop ESRD and need renal replacement therapy (126). More AAV patients receive hemodialysis than peritoneal dialysis, and the relapse rate of AVV in dialyzed patients is low (0.08 episodes per person-year) (175). It is still controversial whether to continue immunosuppressive maintenance therapy in patients on dialysis. Continuing the therapy raises the risk of infectious diseases (175), while the withdrawal may expose the patients to extra-renal manifestations. In MPO-ANCA diseases without extrarenal manifestations, the withdrawal of the maintenance treatment after 6 months seems to be a safe strategy (126). The recent KDIGO guideline suggests discontinuing immunosuppressive therapy after 3 months in patients on dialysis and without any extrarenal manifestation (Practice point) (43). However, a randomized controlled trial needs to be conducted to address this question, such as the ongoing MASTER ANCA trial (NCT03323476).

Renal transplantation provides better quality of life for patients with ESRD. Patients and transplant kidney survival is similar to nondiabetic patients (176). The relapse rate is low (it was 0.02–0.028 episodes per patient-year in a multicenter retrospective cohort) and was not affected by the ANCA subtype or the duration of remission before transplantation (177, 178). The current KDIGO guideline suggests withholding transplantation until the patient is in complete clinical remission for ≥6 months. Persistence of elevated but stable ANCA titers should not delay transplantation (43). Relapse should be confirmed by histopathology, and the same treatment protocol is recommended for the relapse as discussed above (43).

## Upcoming and Possible Therapeutic Targets

Bortezomib is a proteasome inhibitor, which is implicated to inhibit NF-kB, halting cell proliferation, and committing B-cells to apoptosis (179). Bortezomib proved to be efficient in a mouse model of MPO-ANCA glomerulonephritis (180). A case study reported complete remission in a refractory disease (181). However, clinical trials have not been performed because of its frequent adverse events (182, 183).

Adding hydroxychloroquine to standard therapy proved to be effective in reducing relapses, and steroid doses. It helped to improve symptoms and it was well tolerated as well (184). An ongoing randomized-controlled study (HAVEN) is assessing its efficacy (NCT04316494).

Belimumab, an anti-BAFF monoclonal antibody may inhibit the AAV-associated overexpression of BAFF (185) and promote B-cell apoptosis. Thus, the formation of mature, autoreactive B-cells is impeded and class switching and Th1 responses are also altered favorably. Belimumab was studied as a potential maintenance agent, but it did not reduce the risk of relapse (186).

An ongoing study (COMBIVAS) evaluates the efficacy of combined rituximab and belimumab (NCT03967925), and another one (BREVAS) belimumab combined with azathioprine (186).

Abatacept is a fusion protein of the Fc region of human IgG1 and the extracellular domain of the cytotoxic T-lymphocyte-associated protein 4 (CTLA-4/CD152). CTLA-4 is a constitutively expressed receptor on regulatory T-cells, and it is homologous to the co-stimulatory T-cell protein, CD28. By binding to CD80 and CD86 on antigen-presenting cells with a higher affinity than CD28, abatacept prevents T-cell activation (187). A study proved abatacept effective and well-tolerated in relapsing GPA patients (188), and the results of a double-blind randomized controlled trial of abatacept as first-line treatment for AAV (ABAVAS) are expected to be published soon (NCT00482066). It is to note that these studies excluded patients with severe disease, including glomerulonephritis.

A case report of two patients demonstrated remission with the use of C5 inhibitor, eculizumab, but it has never been studied more thoroughly (189).

A randomized study, Alemtuzumab for ANCA Associated Refractory Vasculitis (ALEVIATE), studied alemtuzumab, a monoclonal antibody against the CD52 on T-cells, targeting them to destruction (190). Although the majority of patients reached remission at 6 months, adverse effects were more common than in standard therapies. Alemtuzumab also triggered vasculitis in several case studies (191, 192).

Tocilizumab, a humanized interleukin-6 receptor antibody, could be a potential therapeutic drug in AAV. The efficacy of tocilizumab has already been proved for large-vessel vasculitis, and some studies also detected clinical remission in AAV (193). Large-scale clinical trials, like SATELITE (NCT04871191), are needed to determine the long-term renal and patient outcomes (33).

Promising results have recently been published from the European IXCHANGE study of an anti-C5a antibody, vilobelimab. Vilobelimab proved to be efficient in maintaining therapy in terms of remission with good safety and tolerability profile. Vilobelimab use is suspected to be able to lower glucocorticoid dosage and its toxicity (NCT03712345).

The pivotal role of peptidylarginin-deiminase 4 (PADI) in NET formation has been demonstrated. Correspondingly, PADI inhibitors reduce histone citrullination and NET release both *in vivo* and *in vitro* (194). Selective PADI-4 inhibitor, GSK484 was successful in anti-MPO glomerulonephritis by reducing the inflammatory response and the subsequent segmental necrosis (195). Hence, PADI inhibitors could potentially contribute to the therapeutic armamentarium of NETosis-associated diseases.

Leflunomide is a dihydro-orotate-dehydrogenase inhibitor which is responsible for pyrimidine synthesis. By halting the synthetic pathway, it arrests the actively proliferating T-, and B-lymphocytes and proved efficient in a small number of patients (196). Currently, a study is comparing the efficacy of azathioprine and leflunomide as a maintenance regime (NCT04737343).

**Figure 4:**
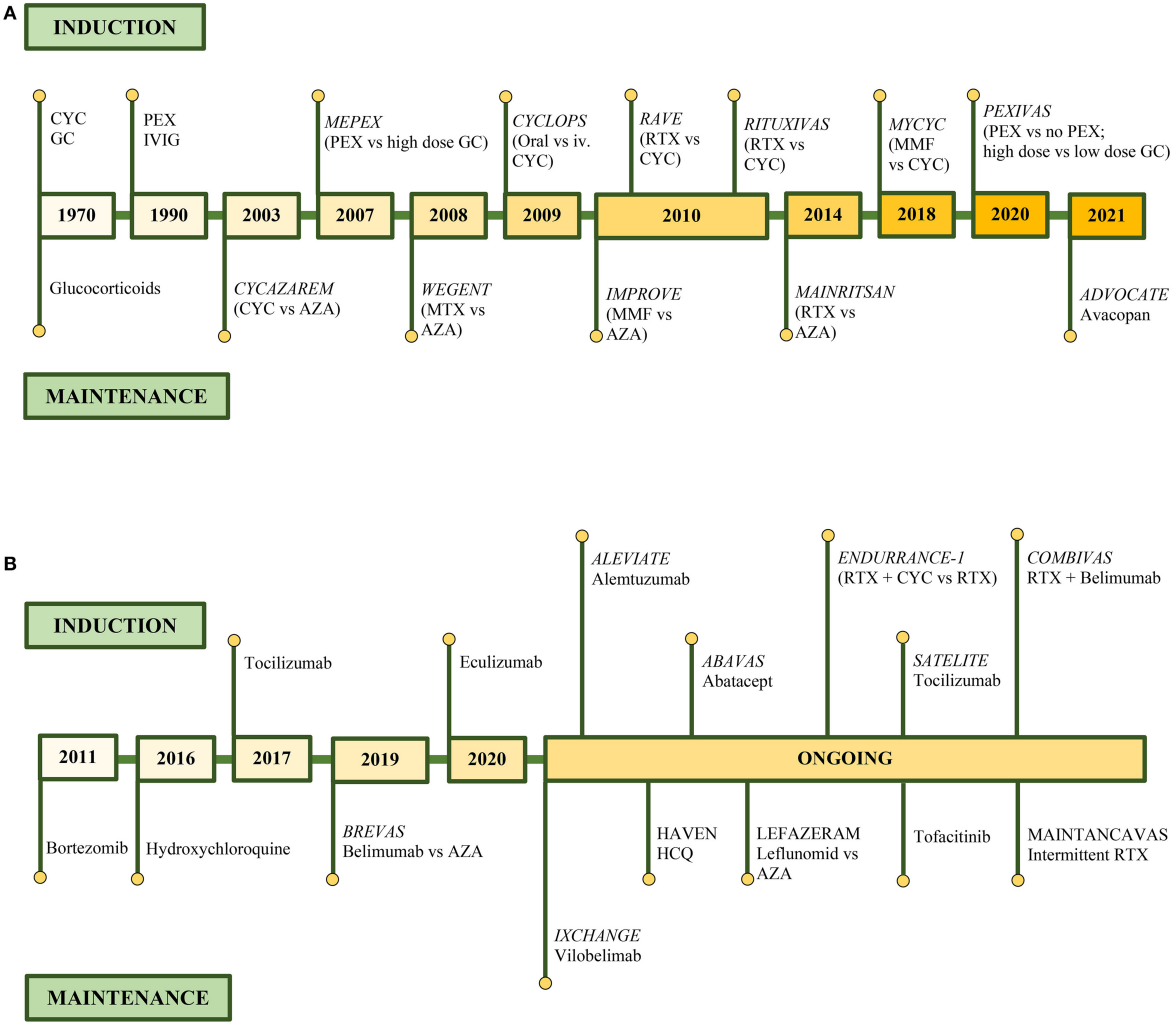
Therapeutic evolution of ANCA-associated glomerulonephritis. Clinical trials in remission induction and maintenance therapy of currently used medications **(A)**, and medications with positive outcome but currently not presented in guidelines and on-going clinical trials **(B)**.

Figure 4 summarizes the therapeutic evolution of ANCA-associated glomerulonephritis, with the milestones of the currently used therapies (Figure 4A), and previous trials with medications with positive outcome but not used in practice with ongoing trials (Figure 4B) (181, 184, 186, 189, 193, 197–199) (NCT03712345, NCT04316494, NCT00482066, NCT04737343, NCT00482066, NCT04871191, NCT04973033, NCT03942887, NCT02749292, NCT03967925).

## Conclusions

The initially nonspecific, then heterogenic symptoms can mislead the diagnostic approach and cause substantial delay in the adequate treatment of ANCA-associated glomerulonephritis. In the absence of distinct symptoms, renal-limited and not rapidly progressive forms are also challenging to identify. The clinical features and laboratory parameters of ANCA-associated vasculitis can mimic infection, malignancy, and other autoimmune diseases. ANCA-negative and atypical ANCA cases can complicate the road to the diagnosis. The first but necessary step of the diagnostic approach is to think of it. Although the treatment options are continuing to improve, the prompt diagnosis and treatment initiation of ANCA-associated glomerulonephritis have remained crucial factors in patient and renal outcome.

## Author Contributions

AM, PS, and NL participated in reviewing the literature and writing the manuscript. All authors agree to be accountable for the content of the work.

## Conflict of Interest

The authors declare that the research was conducted in the absence of any commercial or financial relationships that could be construed as a potential conflict of interest.

## Publisher's Note

All claims expressed in this article are solely those of the authors and do not necessarily represent those of their affiliated organizations, or those of the publisher, the editors and the reviewers. Any product that may be evaluated in this article, or claim that may be made by its manufacturer, is not guaranteed or endorsed by the publisher.
